# Serotonin syndrome induced by Bromfed DM in a patient on sertraline

**DOI:** 10.1177/2050313X251392069

**Published:** 2025-11-20

**Authors:** John K. Jung, Ismail Zazay, James R. Burmeister, Neda Shaghaghi

**Affiliations:** 1 John Sealy School of Medicine, University of Texas Medical Branch, Galveston, TX, USA; 2 Department of Foundational Medical Studies, Oakland University William Beaumont School of Medicine, Rochester, MI, USA; 3 Department of Family Medicine, University of Texas Medical Branch, Galveston, TX, USA

**Keywords:** serotonin syndrome, SSRI-dextromethorphan interaction, adverse drug reaction, pharmacovigilance, over-the-counter medication risk

## Abstract

Serotonin syndrome (SS) is a potentially life-threatening condition caused by excessive serotonergic activity in the central nervous system. It is commonly linked to combinations of serotonergic prescription drugs; however, over-the-counter or prescription cold medications containing dextromethorphan (DM) or first-generation antihistamines may also pose a risk. We report a case of a 52-year-old male on sertraline who developed SS after taking Bromfed DM, a cough syrup containing brompheniramine, pseudoephedrine, and DM. Three days after starting the medication, he experienced confusion, dilated pupils, myoclonic foot movements, and difficulty with speech. Symptoms improved following the discontinuation of both sertraline and Bromfed DM. This case underscores the importance of recognizing the serotonergic potential of common cold medications and the risk of SS even at therapeutic doses, particularly in patients on selective serotonin reuptake inhibitors (SSRIs). Prompt identification and cessation of the causative agents are critical to avoid complications.

## Introduction

Serotonin syndrome (SS) is a potentially life-threatening toxidrome caused by excessive serotonergic activity in the central nervous system. Classically, it presents with a triad of altered mental status, autonomic instability, and neuromuscular hyperactivity.^1,2^ Common symptoms include confusion or agitation, diaphoresis, tachycardia, hyperthermia, dilated pupils, and characteristic neuromuscular findings such as tremor, hyperreflexia, and clonus.^3,4^ SS usually arises from drug interactions or overdose involving two or more serotonergic agents that synergistically elevate serotonin levels.^1,5^

While combinations of prescription medications are well-known culprits, prescription cough and cold preparations can also precipitate SS when combined with serotonergic agents.^1,3,4^ This case highlights the risk of SS following the addition of Bromfed dextromethorphan (DM) to a stable selective serotonin reuptake inhibitor (SSRI) regimen, emphasizing the serotonergic potential of common cold medications and the importance of clinician awareness of such drug interactions.

## Case presentation

A 52-year-old male with a medical history of hyperthyroidism, hypertension, and generalized anxiety disorder had been on sertraline 50 mg daily since September 2023. Other medications included propranolol 10 mg daily, methimazole 10 mg daily, and olmesartan-hydrochlorothiazide (HCTZ) 40–25 mg daily.

Four days prior to the office visit, the patient presented to urgent care for a persistent cough. He tested negative for COVID-19, influenza, and streptococcus and was prescribed Bromfed DM syrup (brompheniramine 2 mg, pseudoephedrine 30 mg, dextromethorphan 10 mg per 5 mL) to be taken 5 mL every 6 hours, along with a methylprednisolone dose pack.

Three days after initiating Bromfed DM, the patient developed dizziness, confusion, photophobia, irritability, difficulty with speech articulation, and noticeably dilated pupils. He also reported a sensation of increased eye pressure, which he described as “eye bulging,” that impaired his ability to drive safely. His wife observed spontaneous foot movements during sleep, which were consistent with myoclonic jerks.

The patient and his wife independently researched his symptoms, suspected SS, and discontinued both sertraline and Bromfed DM a day before his primary care appointment.

On examination at that appointment, he reported mild improvement. Vitals were normal (BP: 106/57, HR: 70 bpm, Temp: 36.7°C). He had intact neurological function, normal gait, no hyperreflexia, and no clonus. No laboratory or imaging studies were deemed necessary at this time, as the clinical presentation was consistent with resolving SS and the patient’s condition was improving. However, he continued to experience disorientation and cognitive impairment in the absence of any alcohol use.

## Discussion

SS is mediated primarily through excessive stimulation of central 5-HT₂A receptors.^4^ Clinical features typically emerge rapidly, within hours, of serotonergic agent exposure or dose increase. Symptoms span mental status changes (agitation, confusion), autonomic dysfunction (hyperthermia, blood pressure/heart rate instability), and neuromuscular abnormalities (hyperreflexia, clonus).^2,4^ While SS classically manifests within hours of a precipitating change, more subacute presentations evolving over several days have also been reported. In our patient, symptoms emerged approximately 72 hours after Bromfed DM initiation, illustrating a less typical subacute course.

The patient’s symptom pattern and timeline met key elements of the Hunter Serotonin Toxicity Criteria, which require serotonergic agent use plus any of the following: spontaneous clonus; inducible clonus with agitation or diaphoresis; ocular clonus with agitation or diaphoresis; tremor with hyperreflexia; or hypertonia with temperature > 38°C and ocular or inducible clonus.^2^ In this case, the spontaneous foot movements (suggestive of clonus), dilated pupils, confusion, and irritability occurred in the setting of serotonergic polypharmacy and improved upon drug cessation, consistent with moderate SS.

The interaction is pharmacologically plausible. DM, an NMDA antagonist, has serotonin reuptake inhibition and releasing activity.^6,7^ Sertraline inhibits CYP2D6, which metabolizes DM, potentially raising serum levels.^8^ Brompheniramine, a first-generation antihistamine, also has serotonin reuptake inhibitory properties.^9^ Pseudoephedrine does not affect serotonin but may have exacerbated autonomic symptoms through sympathomimetic mechanisms. None of the patient’s other chronic medications (propranolol, methimazole, olmesartan) have serotonergic activity, and thus they were unlikely to have contributed to this reaction. It is possible that propranolol (a beta-blocker) masked certain autonomic signs of SS (such as tachycardia), but it did not precipitate the syndrome.

Though SS is often associated with overdose or the use of multiple serotonergic agents, the literature supports toxicity at therapeutic doses in susceptible patients.^8,10^ Similar cases have reported SS after combining SSRIs with over-the-counter cold medications such as NyQuil.^10^ This case reinforces the necessity for clinicians to consider such interactions, even with seemingly benign non-prescription or prescription cold formulations. Management of SS is primarily supportive. Our patient improved with withdrawal of the offending agents and did not require any pharmacologic therapy. In general, mild-to-moderate cases resolve with discontinuation of serotonergic drugs and supportive care measures. However, severe SS may necessitate sedation with benzodiazepines, active cooling, and administration of a serotonin antagonist, such as cyproheptadine. Patients with life-threatening features, such as hyperthermia or profound agitation, may require intensive care management, including neuromuscular paralysis and mechanical ventilation, to prevent complications.

## Conclusion

This case illustrates a preventable incident of SS in a patient on stable SSRI therapy who developed symptoms after initiating Bromfed DM, a common cold medication. The combination of DM and brompheniramine, both possessing serotonergic activity, with an SSRI led to a clinically significant reaction.

Clinicians must maintain high vigilance for SS in patients on serotonergic medications who present with new neurologic or autonomic symptoms, particularly when recent medication changes or additions have occurred. Patient education, careful medication reconciliation, and avoidance of serotonergic cold preparations in SSRI users are critical to preventing similar events.

## Supplemental Material

sj-docx-1-sco-10.1177_2050313X251392069 – Supplemental material for Serotonin syndrome induced by Bromfed DM in a patient on sertraline Supplemental material, sj-docx-1-sco-10.1177_2050313X251392069 for Serotonin syndrome induced by Bromfed DM in a patient on sertraline by John K. Jung, Ismail Zazay, James R. Burmeister and Neda Shaghaghi in SAGE Open Medical Case Reports

## References

[1] Boyer EW Shannon M. The serotonin syndrome. N Engl J Med 2005; 352(11): 1112–1120. 15784664 10.1056/NEJMra041867

[2] Dunkley EJ Isbister GK Sibbritt D , et al The Hunter Serotonin Toxicity Criteria: simple and accurate diagnostic decision rules for serotonin toxicity. QJM 2003; 96(9): 635–642. 12925718 10.1093/qjmed/hcg109

[3] Schwartz AR Pizon AF Brooks DE. Dextromethorphan-induced serotonin syndrome. Clin Toxicol 2008; 46(8): 771–773. 10.1080/15563650701668625 19238739

[4] Francescangeli J Karamchandani K Powell M , et al The serotonin syndrome: from molecular mechanisms to clinical practice. Int J Mol Sci 2019; 20(9): 2288. 31075831 10.3390/ijms20092288 PMC6539562

[5] Monte AA Chuang R Bodmer M. Dextromethorphan, chlorpheniramine and serotonin toxicity. Br J Clin Pharmacol 2010; 70(6): 794–798. 21175434 10.1111/j.1365-2125.2010.03747.x PMC3014062

[6] Dy P Arcega V Ghali W , et al Serotonin syndrome from escitalopram and dextromethorphan. BMJ Case Rep 2017; 2017: bcr2017221486. 10.1136/bcr-2017-221486 PMC5747823 28784915

[7] Foong AL Grindrod KA Patel T , et al Demystifying serotonin syndrome (or serotonin toxicity). Can Fam Physician 2018; 64(10): 720–727. 30315014 PMC6184959

[8] Simon LV Torrico TJ Keenaghan M. Serotonin syndrome. In: StatPearls [Internet]. StatPearls Publishing, 2024. 29493999

[9] Gillman PK. Serotonin syndrome: history and risk. Fundam Clin Pharmacol 1998; 12(5): 482–491. 9794145 10.1111/j.1472-8206.1998.tb00976.x

[10] Sethi R Kablinger AS Kavuru B. Serotonin syndrome in a sertraline-treated man taking NyQuil. Prim Care Companion CNS Disord 2012; 14(6): PCC.12l01388. 10.4088/PCC.12l01388 PMC3622531 23585992

