# Supplementary material for: Serotonin syndrome induced by Bromfed DM in a patient on sertraline
Source: SAGE Open Med Case Rep. 2025 Nov 20;13:2050313X251392069. doi: 10.1177/2050313X251392069 (PMC12638710; doi:10.1177/2050313X251392069)
Supplement: sj-docx-1-sco-10.1177_2050313X251392069 – Supplemental material for Serotonin syndrome induced by Bromfed DM in a patient on sertraline [file sj-docx-1-sco-10.1177_2050313X251392069.docx]

**Appendix: CARE Checklist**

| **CARE Checklist Item** | **Presence in Manuscript** |
| --- | --- |
| Title: the title includes “case report” | Yes – “Serotonin Syndrome Induced by Bromfed DM in a Patient on Sertraline: A Case Report.” |
| Key words: 2–5 keywords (incl. “case report”) | Yes – Serotonin Syndrome; SSRI–Dextromethorphan Interaction; Adverse Drug Reaction; Pharmacovigilance; Over-the-Counter (OTC) Medication Risk. |
| Abstract: summary of case (unstructured) | Yes – Unstructured ~150-word abstract summarizing background, presentation, interventions (drug cessation), outcome, and key lesson. |
| Introduction: why the case is unique; context | Yes – Highlights SS precipitated by an OTC cold medication added to stable SSRI; situates within literature. |
| Patient Information: de-identified info, history | Yes – 52-year-old male; hyperthyroidism (methimazole), hypertension (olmesartan-HCTZ), GAD (sertraline 50 mg); chief complaint: cough treated with Bromfed DM; no contributory family/psychosocial history. |
| Clinical Findings: important exam & findings | Yes – After 3 days of Bromfed DM: confusion, disorientation, photophobia, irritability, impaired speech, dilated pupils, myoclonic jerks during sleep; vitals normal; no persistent clonus/hyperreflexia; residual cognitive impairment. |
| Timeline: patient’s clinical course | Yes – Day 0: Bromfed DM prescribed; Days 1–3: took medication; Day 3: SS symptoms; late Day 3: stopped Bromfed DM + sertraline; Day 4: clinic evaluation with improvement. |
| Diagnostic Assessment: methods, challenges, differential | Yes – Clinical diagnosis consistent with Hunter Criteria; no labs/imaging given classic presentation and clinical improvement; differentials (infection, stroke, metabolic issues) considered unlikely. |
| Therapeutic Intervention: type, dosage, duration | Yes – Immediate cessation of sertraline and Bromfed DM; supportive care (observation, reassurance, hydration). No cyproheptadine or other pharmacologic therapy required. |
| Follow-up & Outcomes: outcomes, adherence, adverse events | Yes – Symptoms improved within 24–48 h; complete resolution over subsequent days; no sequelae, no adverse events; educated to avoid serotonergic cold meds; no recurrence. |
| Discussion: literature, strengths, limitations, conclusions | Yes – Mechanism of SSRI–dextromethorphan/brompheniramine interaction; recognizes therapeutic-dose risk; notes typical rapid onset and possible subacute course; outlines management for mild–severe SS; clear take-home message on OTC risk. |
| Patient Perspective | No (not provided) – Manuscript notes the patient and spouse suspected SS and acted appropriately; they were satisfied with outcome and educated on avoidance. |
| Informed Consent: consent for publication | Yes – Written informed consent obtained and stated in Ethics/Consent section. |
